# COVID-19 information sources, knowledge, attitude, control practices and the predictors among health workers during the pandemic in Ebonyi state, Nigeria

**DOI:** 10.1038/s41598-024-57647-1

**Published:** 2024-03-25

**Authors:** Ugwu I. Omale, Ifeyinwa M. Okeke, Okechukwu O. Ukpabi, Richard L. Ewah, Osarhiemen Iyare, Chidinma I. Amuzie, Onyinyechukwu U. Oka, Azuka S. Adeke, Victor U. Uduma, Glory E. Nkwo, Cordis O. Ikegwuonu, Ugochi I. A. Nwali, Olaedo O. Nnachi

**Affiliations:** 1 Department of Community Medicine, Alex Ekwueme Federal University Teaching Hospital Abakaliki (AEFUTHA), Abakaliki, Ebonyi State Nigeria; 2 Department of Anaesthesia, Alex Ekwueme Federal University Teaching Hospital Abakaliki (AEFUTHA), Abakaliki, Ebonyi State Nigeria; 3 Anaesthesia Unit, Department of Surgery, Ebonyi State University, Abakaliki, Ebonyi State Nigeria; 4 Department of Community Medicine, Federal Medical Centre Umuahia, Umuahia, Abia State Nigeria; 5 Department of Internal Medicine, Alex Ekwueme Federal University Teaching Hospital Abakaliki (AEFUTHA), Abakaliki, Ebonyi State Nigeria

**Keywords:** COVID-19, Information sources, Knowledge, Attitude, Control practices, Predictors, Health workers, Nigeria, Medical research, Epidemiology, Infectious diseases

## Abstract

The COVID-19 pandemic has changed into an endemic COVID-19 disease and health workers continue to be at high risk. The situation requires continued use of COVID-19 control measures by health workers and this will likely depend on their sources of information/knowledge/attitude about COVID-19 and previous use of COVID-19 control measures. We explored the COVID-19 information sources, knowledge, attitude, control practices, and the predictors, among health workers in Ebonyi state, Nigeria. We implemented an online-offline analytical cross-sectional survey from March 12 to May 9, 2022 among all categories of health workers (clinical/non-clinical, public/private) working/living in Ebonyi state who gave consent and were selected via convenience/snowballing sampling. Data was collected with a structured self-administered/interviewer-administered questionnaire via WhatsApp/KoBoCollect. Descriptive/inferential analyses were done including multivariate generalized linear models. 1276 health workers were surveyed. The commonest individual source of information about COVID-19 was health workers (used by 83.8%), followed by radio (67.9%), television (59.6%), family members/relatives/friends (57.9%) etc. The main individual source of information for majority of the participants was health workers (for 35.0%) followed by radio (24.5%), television (14.4%) etc. The most trusted individual source of information for majority of the participants was health workers (for 39.4%) followed by radio (26.0%), television (14.3%) etc. Interpersonal sources were the main/most trusted source of information for the majority (48.0%/49.8%) followed by traditional media (39.4%/40.6%) and internet/social media/SMS (12.6%/9.6%). 42.3%, 81.3%, and 43.0% respectively had good knowledge, good attitude, and good control practice about COVID-19. The most important predictors of the main/most trusted sources of information about COVID-19 were place of work (public/private), level of place of work (primary-secondary/tertiary), age, and years of working experience. Good knowledge about COVID-19, good attitude towards COVID-19, strong COVID-19 experience/perception, working at a tertiary facility, tertiary education, and decrease in years of working experience were strong predictors of good control practice about COVID-19. This study’s evidence regarding the commonest/main/most trusted information sources and control practice about COVID-19 should be considered by later COVID-19/similar health emergencies’ policy actions to optimise emergency health information dissemination and use of control measures by health workers in Ebonyi state/Nigeria/other similar settings.

## Introduction

After the devastating global health and economic effects of the coronavirus disease 2019 (COVID-19) pandemic since the emergence of COVID-19 more than four years ago^1^, COVID-19 has been declared to no longer be a public health emergency of international concern^2^. However, as an endemic disease with persistence of new infections and re-infections and the risk of upsurge from new strains of the COVID-19 virus, COVID-19 still poses a real threat to the health and economies of populations around the world^1^. Notwithstanding the decrease in the testing and reporting rates (and the fact that testing and reporting have stopped in many countries)^1,3^, more than 503,000 COVID-19 cases and over 10,000 related deaths were confirmed between the 28-day period of 7 January and 4 February 2024^3^. The non-use or decline in the use of preventive public health measures have been contributing to the persistence of the threat from COVID-19 and it is thus global imperative to continue the use of COVID-19 control measures for the foreseeable future^1^.

Health workers were at higher risk of contracting COVID-19 compared to the general public because of their involvement in management of patients, including COVID-19 patients, and the risk persisted as new COVID-19 infections or re-infections continued to occur. Hence, the continued use of COVID-19 control measures by health workers was very essential and would very likely be influenced by not only their COVID-19 health information sources, level of knowledge, and level of attitude towards COVID-19^4–6^, but also by their level of use of these control measures during the COVID-19 pandemic.

The knowledge, attitude, and practices about COVID-19 among health workers during the pandemic were assessed by some studies around the world and in Nigeria^4–17^. Fewer studies assessed health workers’ sources of information about COVID-19^6,7,9–15^. However, most of these studies were only online studies among limited categories of health workers and carried out during the initial waves of the pandemic. We could not identify any studies that extensively assessed health workers’ use and trust for COVID-19 information sources and the determinants. To our knowledge, there was no study on health workers’ information sources, knowledge, attitude, and practices about COVID-19 during the pandemic in Ebonyi state.

Context-specific understanding of how the health workers in Ebonyi state, Nigeria, were getting information about COVID-19 and their knowledge, attitude, and control practices, including the predictors, would guide subsequent public health policy interventions to enhance the use of preventive public health measures for long-term control of COVID-19 and for the control of any similar infectious diseases/pandemics in the future. The misinformation/disinformation and conspiracy theories about COVID-19 in the social and conventional media was unprecedented. So, it would be particularly important to understand health workers’ use and confidence in the sources of information about COVID-19. In addition, as health opinion leaders and sources of health information for the general public, health workers’ use and confidence in COVID-19 information sources and their knowledge, attitude, and control practices about COVID-19 would most likely influence the general public’ confidence and use of COVID-19 information sources and control practices. An exploratory study of health workers’ use/confidence in COVID-19 information sources and their knowledge, attitude, and control practices was thus an imperative.

We conducted an extensive online and offline study to assess COVID-19 vaccination acceptance and the determinants among the health workers in Ebonyi state^18^. As part of the study, we also explored the COVID-19 information sources, knowledge, attitude, and control practices, and the predictors, among the health workers during the COVID-19 pandemic in Ebonyi state, Nigeria, and the findings are reported in this paper.

## Methods

### Study design and participants

The study was an analytical cross-sectional survey implemented between March 12 and May 9, 2022 among all categories of health workers, including clinical and non-clinical and public and private health workers, in Ebonyi state, southeast of Nigeria. The study protocol has been described elsewhere^18^. Health workers who were working or living in Ebonyi state and gave verbal consent were eligible to participate in the survey. Participants were selected via convenience and snowballing sampling methods. The investigators first contacted many health workers who were available and/or easily accessible physically or via phone calls and sought their consent. Afterwards, those who gave verbal consent were sent the web link for the self-administered electronic questionnaire through their private WhatsApp addresses. They were also asked to forward the web link to other eligible health workers. Health workers who were available and/or easily accessible but had no WhatsApp addresses responded to the electronic questionnaire in android devices after giving verbal consent (to the interviewers). A sample size of I880 was estimated for the parent study^18^ and 1276 (67.9%) of health workers successfully participated in the survey.

### Independent factors and outcome measures

Independent factors include sociodemographic characteristics (gender, age, marital status, educational level) and work-related attributes (work category, years of working experience, primary place of work, level of primary place of work). The outcome measures include the main and most trusted sources of information about COVID-19, level of knowledge of COVID-19, level of attitude towards COVID-19 (vaccination), and level of control practice about COVID-19. Regarding the evaluation of predictors of the level of control practice about COVID-19, additional independent factors include the main and most trusted sources of information about COVID-19, level of knowledge of COVID-19, level of attitude towards COVID-19 (vaccination), and extent of COVID-19 experience and perception.

The sources of information about COVID-19 as well as the main and most trusted sources of information were grouped into interpersonal information sources consisting of family members/relatives/friends, health workers, place of work, place of worship/religious forums; traditional media consisting of radio, television, and prints (newspaper and magazine); and internet/social media/SMS consisting of internet sites, WhatsApp, Facebook, and SMS/text messages.

The assessment of basic knowledge of COVID-19 involved participants responses to 44 knowledge items in the questionnaire. A score of “1” was assigned for each correct response and “0” for each incorrect response, giving a maximum score of 44 and a minimum of zero for each participant. For each participant, a total knowledge score of ≥ 75% of 44 was categorized as good knowledge and < 75% was poor knowledge. The assessment of attitude towards COVID-19 and COVID-19 vaccination involved participants responses to 16 attitude items in the questionnaire. Every attitude item had five options of strongly disagree, disagree, not sure, agree, and strongly agree and scores of “1” to “5” or “5” to “1” were respectively assigned to the items as appropriate, giving a maximum score of 80 and a minimum of 16 for each participant. For each participant, a total attitude score of ≥ 75% of 80 was categorized as good attitude and < 75% was poor attitude. The assessment of control practices about COVID-19 involved participants responses to 24 practice items in the questionnaire. A score of “1” was assigned for each correct response and “0” for each incorrect response, giving a maximum score of 24 and minimum of zero for each participant. For each participant, a total practice score of ≥ 75% of 24 was categorized as good control practice and < 75% was poor control practice.

### Data collection

Data collection was through a structured self-administered and interviewer-administered questionnaire survey of the health workers^18^. The following sections were part of the questionnaire: sociodemographic characteristics; COVID-19 experiences and perceptions; basic knowledge of COVID-19; attitude towards COVID-19 and COVID-19 vaccination; and practices about COVID-19. The electronic version of the questionnaire was programmed using the KoBoToolbox software and was pre-tested among health workers who were not included in the survey. The web link for the electronic questionnaire was distributed to health workers via WhatsApp and they were also asked to forward the web link to other eligible health workers they know within the study area. Interviewer-administered questionnaire in KoBoCollect installed in android devices was used for the survey of health workers who had no WhatsApp addresses and those living in remote/rural areas with very poor or no internet access.

### Statistical analyses

Statistical analyses were carried out using Stata/SE version 15.1 (Stata Corp, College Station, TX, USA). Data was described using frequencies with proportions/percentages and median with inter-quartile range as appropriate. Inferential analyses involved the use of generalized linear models (GLM) at 2.5% significance level to compensate for multiple comparisons. For dichotomous/categorical independent factors, prevalence difference in the outcomes with the corresponding 97.5% CI and p-values were computed using binomial identity GLM models with robust standard errors. For continuous independent factors, coefficients in the outcomes with the corresponding 97.5% CI and p-values were computed using the binomial identity GLM models. All the independent variables were simultaneously added to the GLM model in the adjusted analyses. Where binomial identity GLM models failed to achieve convergence, Gaussian identity GLM models were used instead^19^.

### Ethics approval and consent to participate

Ethical approval for this study was obtained from the Ebonyi State Health Research and Ethics Committee (EBSHREC/15/01/2022-02/01/2023) and Research and Ethics Committee of Alex Ekwueme Federal University Teaching Hospital Abakaliki (14/12/2021–17/02/2022). Verbal informed consent was obtained from the study participants during which the purpose the study, kind of participation, likely duration of participation, voluntary nature of participation, absence of potential harm, potential benefit, and confidential nature of the study were duly communicated to them. The research procedures were in compliance with the Declaration of Helsinky.

## Results

### Sociodemographic and background characteristics

The sociodemographic and background (work-related) characteristics of the study participants are presented in Table 1. The median age (IQR) was 33 years (26–43) and the median years of working experience was 5 years (2–13); 857 (67.2%) were females; 691 (54.2%) were married; 726 (56.9%) had a tertiary education; 1110 (87.0%) were clinical staff; 652 (51.1%) were working primarily in private health facilities; and 952 (74.6%) were working at primary health facilities.

**Table 1:** Sociodemographic and background characteristics of the 1276 study participants.

	n	%
Gender		
Male	419	32.8
Female	857	67.2
Age, median (IQR), years	33 (26–43)	–
Marital status		
Married	691	54.2
Not married^1^	585	45.8
Educational level		
No formal education	10	0.8
Primary	36	2.8
Secondary	504	39.5
Tertiary	726	56.9
Work category or cadre		
Non-clinical staff^2^	166	13.0
Clinical staff^3^	1110	87.0
Working experience, median (IQR), years	5 (2–13)	–
Primary place of work		
Private health facility^4^	652	51.1
Public health facility^5^	624	48.9
Level of primary place of work		
Primary health facility^6^	952	74.6
Secondary health facility^7^	39	3.1
Tertiary health facility^8^	285	22.3

^1^Separated or Divorced or Widowed or Never married (Single).

^2^Admin, Personnel, Account, PRO, Security etc.

^3^Patent medicine vendor, Primary health care worker (Health attendant, Community health extension worker, Community health officer, Nurse & midwife), Orderly, Medical laboratory scientist or technologist, Pharmacist or pharmacy technician, Medical doctor, and others (Dental therapist, physiotherapist, Dietician etc.).

^4^Patent medicine vendor (PMV), Private pharmacy, Private laboratory, Private hospital or clinic, Missionary hospital.

^5^Primary health care (PHC) centre, General hospital, Federal tertiary health centre, and Federal university teaching hospital.

^6^PMV, Private pharmacy, Private laboratory, Private hospital or clinic, and PHC centre.

^7^Missionary hospital and General hospital.

^8^Federal tertiary health centre and Federal university teaching hospital.

### Sources of information about COVID-19

The study participants’ sources of information about COVID-19, including their main and most trusted sources, are presented in Table 2. For interpersonal sources, the commonest source of information was health workers (used by 1069 or 83.8% of participants) followed by family members/relatives/friends (used by 739 or 57.9%), place of work (used by 606 or 48.3%), and place of worship (used by 577 or 45.2%). For traditional media sources, the commonest source of information was radio (used by 867 or 67.9%) followed by television (used by 760 or 59.6%) and prints (used by 336 or 26.3%). For internet/social media/text message sources, the commonest source of information was WhatsApp (used by 412 or 32.3%) and text messages (used by 412 or 32.3%) followed by Facebook (used by 407 or 31.9%) and internet sites (used by 394 or 30.9%). Overall, the commonest source of information was health workers (used by 83.8%) followed by radio (used by 67.9%), television (used by 59.6%), family members/relatives/friends (used by 57.9%), etc. (Table 2).

**Table 2:** Sources of information about COVID-19 among the 1276 study participants.

	n	%
Interpersonal		
Health workers	1069	83.8
Family members/relatives/friends	739	57.9
Place of work	606	48.3
Place of worship/Religious forums	577	45.2
Traditional media		
Radio	867	67.9
Television	760	59.6
Prints (Newspaper/Magazine)	336	26.3
Internet, social media, & SMS*		
WhatsApp	412	32.3
Text messages*	412	32.3
Facebook	407	31.9
Internet sites	394	30.9
Main source of information		
Interpersonal	613	48.0
Health workers	447	35.0
Family members/Relatives/Friends	99	7.8
Place of work	51	4.0
Place of worship/Religious forums	16	1.2
Traditional media	502	39.4
Radio	312	24.5
Television	184	14.4
Prints (Newspaper/Magazine)	6	0.5
Internet, social media, & SMS*	161	12.6
Internet sites	101	7.9
Facebook	38	3.0
WhatsApp	17	1.3
Text messages*	5	0.4
Most trusted source of information		
Interpersonal	636	49.8
Health workers	503	39.4
Family members/Relatives/Friends	63	4.9
Place of work	53	4.2
Place of worship/Religious forums	17	1.3
Traditional media	518	40.6
Radio	332	26.0
Television	183	14.3
Prints (Newspaper/Magazine)	3	0.2
Internet, social media, & SMS*	122	9.6
Internet sites	97	7.6
Facebook	13	1.0
WhatsApp	10	0.8
Text messages*	2	0.2

*Such as text messages or bulk SMS from the Nigerian Centre for Disease Control, National Primary Health Care Development Agency, Banks etc.

The main sources of information for majority of the participants were interpersonal sources (for 613 or 48.0%) followed by traditional media (for 502 or 39.4%) and internet/social media/text messages (for 161 or 12.6%). The main individual source of information was health workers (for 447 or 35.0%) followed by radio (for 312 or 24.5%), television (for 184 or 14.4%) etc. (Table 2). The most trusted sources of information for majority of the participants were interpersonal sources (for 636 or 49.8%) followed by traditional media (for 518 or 40.6%) and internet/social media/text messages (for 122 or 9.6%) (Table 2). The most trusted individual source of information for majority of the participants was health workers (for 503 or 39.4%) followed by radio (for 332 or 26.0%), television (for 183 or 14.3%) etc. (Table 2).

### Knowledge of COVID-19

The study participants’ knowledge of COVID-19 is presented in Table 3. Most of the participants (1253 or 98.2%) knew people get COVID-19 by staying close to infected persons when they cough or sneezes. The most reported symptom of COVID-19 was cough (by 1112 or 87.2% of participants) followed by fever (by 1082 or 84.8%), tiredness (by 806 or 63.2%), difficulty in breathing (by 792 or 62.1%), sore throat (by 683 or 53.5%), body pains (by 644 or 50.5%), chest pain (by 563 or 44.1%), headache (by 553 or 43.3%), etc. (Table 3). Elderly people were said to be more at risk of having severe COVID-19 by 1097 or 86.0% of the participants, 592 (46.4%) said people with chronic illness, 379 (29.7%) said young adults, 324 (25.4%) said children, 257 (20.1%) said people who smoke, 202 (15.8%) said pregnant women etc. (Table 3). Regarding COVID-19 preventive measures: 1244 (97.5%) said by wearing of face mask, 1240 (97.2%) said by maintaining at least 1–2 m distance away from people coughing or sneezing, 1217 (95.4%) said by avoiding crowd, 1189 (93.2%) said by frequent washing of hands with soap and water, etc. (Table 3).

**Table 3:** Knowledge of COVID-19 among the 1276 study participants.

	n	%		n	%
What is COVID-19?			Are there treatments for COVID-19?		
A new type of coronavirus disease	1184	92.8	Yes	956	74.8
An old type of coronavirus disease	63	4.9	No	204	16.0
Do not know	29	2.3	Do not know	118	9.2
How do people get COVID-19?			Are there vaccines for COVID-19?		
By staying close to infected persons when they cough or sneezes	1253	98.2	Yes	1252	98.1
Do not know	16	1.2	No	4	0.3
Others ways*	7	0.6	Do not know	20	1.6
What is the incubation period of COVID-19?			Do you know any COVID-19 vaccination place?		
2–14 days (within 2 weeks)	1055	82.7	Yes	1208	94.7
2–4 weeks	51	4.0	No	44	3.4
> 4 weeks	17	1.3	Who are more at risk of having severe COVID-19? (Multiple response)		
Do not know	153	12.0	Elderly people	1097	86.0
Symptoms of COVID-19 are? (Multiple response)			People with chronic illness	592	46.4
Cough	1112	87.2	Young adults	379	29.7
Fever	1082	84.8	Children	324	25.4
Tiredness	806	63.2	People who smoke	257	20.1
Difficulty in breathing	792	62.1	Pregnant women	202	15.8
Sore throat	683	53.5	Obese people	98	7.7
Body aches/pains	644	50.5	Slim people	52	4.1
Chest pain	563	44.1	Do not know	89	7.0
Headache	553	43.3	How to prevent COVID-19? (Multiple response)		
Loss of taste or smell sensation	403	31.6	Wearing of face mask	1244	97.5
Nausea or vomiting	360	28.2	Maintaining at least 1–2 m distance away from people coughing or sneezing	1240	97.2
Diarrhoea	287	22.5	Avoiding crowd	1217	95.4
Do not know	15	1.2	Frequent hands washing with soap and water	1189	93.2
Can people also have COVID-19 without showing any symptoms?			Frequent hand cleaning with alcoholic sanitisers	1131	88.6
Yes	874	68.5	Avoiding touching of face (eyes, nose, & mouth)	921	72.2
No	246	19.3	COVID-19 vaccination	803	62.9
Do not know	156	12.2	Use of ginger or garlic	86	6.7
Is there a laboratory test to diagnose COVID-19?			Taking chloroquine	85	6.7
Yes	1197	93.8	Use of herbs or roots (native medicines)	30	2.4
No	18	1.4	Taking hot drinks or “ogogoro”^^^	23	1.8
Do not know	61	4.8	Do not know	2	0.2
Where is laboratory test to diagnose COVID-19 done in Ebonyistate? (Multiple response)					
AEFUTHA**	1084	84.9			
General hospitals	208	16.3			
Missionary hospitals	25	2.0			
Primary healthcare centres	15	1.2			
Private laboratories	10	0.8			
Private hospitals	4	0.3			
Do not know	73	5.7			

*From rat, spiritual attack, and bat.

**The Federal University Teaching Hospital in the state.

^^^Local gin.

### Attitude towards COVID-19 and COVID-19 vaccination

The study participants’ attitude towards COVID-19 and COVID-19 vaccination is presented in Table 4. More of the participants (769 or 60.3%) strongly agreed that COVID-19 was real followed by those who agreed (277 or 21.7%); more of them (811 or 63.6%) strongly agreed the risk of getting COVID-19 could be reduced by avoiding crowd followed by those who agreed (325 or 25.5%); more of them (807 or 63.3%) strongly agreed the risk of getting COVID-19 could be reduced by wearing face mask followed by those who agreed (337 or 26.4%); more of them (385 or 30.2%) strongly disagreed the risk of getting COVID-19 could be reduced by taking chloroquine followed by those who were not sure (375 or 29.4%); more of them (414 or 32.4%) strongly disagreed the risk of getting COVID-19 could be reduced by the use of herbs/roots (native medicine) followed by those who were not sure (393 or 30.8%); etc. (Table 4).

**Table 4:** Attitude towards COVID-19 and COVID-19 vaccination among the 1276 study participants.

	Strongly disagree n (%)	Disagree n (%)	Not sure n (%)	Agree n (%)	Strongly agree n (%)
COVID-19 is real	167 (13.1)	15 (1.2)	48 (3.7)	277 (21.7)	769 (60.3)
COVID-19 a serious illness that can kill	103 (8.1)	15 (1.2)	40 (3.1)	300 (23.5)	818 (64.1)
Everybody is susceptible to COVID-19 infection	100 (7.8)	53 (4.2)	87 (6.8)	390 (30.6)	646 (50.6)
The risk of getting COVID-19 can be reduced:					
By avoiding crowd	95 (7.4)	17 (1.3)	28 (2.2)	325 (25.5)	811 (63.6)
By maintaining at least 1–2 m distance away from people coughing or sneezing	89 (7.0)	10 (0.8)	35 (2.7)	410 (32.1)	732 (57.4)
If everybody covers the mouth and nose (with handkerchief or bent elbow) when coughing or sneezing	86 (6.7)	19 (1.5)	41 (3.2)	413 (32.4)	717 (56.2)
By wearing face masks	79 (6.2)	22 (1.7)	31 (2.4)	337 (26.4)	807 (63.3)
By washing hands with soap and water frequently	87 (6.8)	14 (1.1)	31 (2.4)	374 (29.3)	770 (60.4)
By cleaning hands with alcoholic sanitisers frequently	77 (6.0)	19 (1.5)	33 (2.6)	346 (27.1)	801 (62.8)
Chloroquine is effective for treatment/prevention of COVID-19	385 (30.2)	295 (23.1)	375 (29.4)	126 (9.9)	95 (7.4)
Herbs and roots (native medicine) are effective for treatment/prevention of COVID-19	414 (32.4)	406 (31.8)	393 (30.8)	39 (3.1)	24 (1.9)
Ginger and garlic are effective for treatment/prevention of COVID-19	411 (32.2)	424 (33.2)	330 (25.9)	82 (6.4)	29 (2.3)
Hot drinks or “ogogoro”* is effective for treatment/prevention of COVID-19	622 (48.8)	345 (27.0)	266 (20.8)	25 (2.0)	18 (1.4)
COVID-19 vaccines are safe for people to receive	104 (8.2)	74 (5.8)	178 (13.9)	375 (29.4)	545 (42.7)
The risk of COVID-19 can be reduced by receiving COVID-19 vaccination	84 (6.6)	50 (3.9)	123 (9.6)	421 (33.0)	598 (46.9)
Everybody should receive the recommended COVID-19 vaccination	91 (7.1)	86 (6.7)	119 (9.3)	371 (29.1)	609 (47.7)

*Local gin.

### Control practices about COVID-19

The study participants’ control practices about COVID-19 is presented in Table 5. The most ever practiced preventive measure was the wearing of face mask (by 1176 or 92.2%) followed by maintaining at least 1–2 m distance away from people coughing or sneezing (by 1078 or 84.5%), frequent washing of hands with soap and water (by 1045 or 81.9%) and frequent cleaning of hands with alcoholic sanitisers (by 1045 or 81.9%), etc.; and the most currently practiced (in the two weeks preceding the survey) preventive measure was wearing of face mask (by 944 or 74.0%) followed by frequent washing of hands with soap and water (by 728 or 57.1%), frequent cleaning of hands with alcoholic sanitisers (by 654 or 51.3%), the maintaining at least 1–2 m distance away from people coughing or sneezing (by 397 or 31.1%), etc. (Table 5). Taking of chloroquine was the most ever practiced (by 106 or 8.3%) and currently practiced (by 80 or 6.3%) COVID-19 treatment/preventive measure followed by the use of ginger/garlic (by 92 or 7.2% and 51 or 4.0%), etc. Most of the participants (1084 or 85.0%) had never practiced and most of them (1147 or 89.9%) were not currently practicing these treatment/preventive measures (Table 5).

**Table 5:** Control practices about COVID-19 among the 1276 study participants.

	n	%		n	%
Ever practiced the following to prevent COVID-19?			Practicing the following to prevent COVID-19?^^^		
Wearing of a face mask	1176	92.2	Wearing of a face mask	944	74.0
Maintaining at least 1–2 m distance away from people coughing or sneezing	1078	84.5	Frequent hand washing with soap and water	728	57.1
Frequent hand washing with soap and water	1045	81.9	Frequent hand cleaning with alcoholic sanitisers	654	51.3
Frequent hand cleaning with alcoholic sanitisers	1045	81.9	Maintaining 1–2 m from people coughing etc	397	31.1
Avoiding crowd	1007	78.9	Avoiding crowd	387	30.3
Covering your mouth/nose (with handkerchief or your bent elbow) when coughing or sneezing	764	59.9	Covering your mouth/nose (with handkerchief or your bent elbow) when coughing or sneezing)	331	25.9
Avoiding touching your face (eyes, nose, mouth)	731	57.3	Avoiding touching your face (eyes, nose, mouth	270	21.2
Use of bleach/alcohol to clean surfaces	532	41.7	Use of bleach/alcohol to clean surfaces	269	21.1
None of the above was ever practiced	11	0.9	Not practicing any of the above	86	6.7
Ever practiced the following to treat or prevent COVID-19?			Practicing the following to treat or prevent COVID-19?^		
Taking chloroquine	106	8.3	Taking chloroquine	80	6.3
Using ginger or garlic	92	7.2	Using ginger or garlic	51	4.0
Using hot drinks or “ogogoro”*	19	1.5	Using hot drinks or “ogogoro”*	9	0.7
Using herbs or roots (native medicine)	11	0.9	Using herbs or roots (native medicine)	3	0.2
None of the above was ever practiced	1084	85.0	Not practicing any of the above	1147	89.9

*Local gin.

^^^Practicing in the two weeks preceding the survey.

### Level of knowledge, attitude, and control practice about COVID-19 and COVID-19 vaccination

Regarding the level of COVID-19 (vaccination) knowledge, attitude, and control practice among the 1276 study participants: 540 (42.3%) had good knowledge about COVID-19 while 736 (57.7%) had poor knowledge; 1037 (81.3%) had good attitude towards COVID-19 (vaccination) while 239 (18.7%) had poor attitude; 549 (43.0%) had good control practice about COVID-19 while 727 (57.0%) had poor practice.

### Predictors of the main and most trusted sources of information about COVID-19

The associations between the main sources and most trusted sources of information about COVID-19 and sociodemographic and background factors are presented in Table 6. As presented in Table 6, the predictors of interpersonal sources as the main and most trusted source of information about COVID-19 is first presented, with the other sources (traditional media and internet/social media/text message) as reference. The predictors of traditional media as the main and most trusted source of information about COVID-19 is also presented, with the other sources (interpersonal and internet/social media/text message) as reference.

**Table 6:** Association between sociodemographic factors and the main and most trusted sources of information about COVID-19 among the 1276 study participants.

Interpersonal versus non-interpersonal^^^ sources of information						
			Crude results		Adjusted results*	
Main source of information about COVID-19						
	Interpersonal sources n (%)	Not Interpersonal sources^^^ n (%)	cPD (97.5% CI) or cCoef (97.5% CI)	p value	aPD (97.5% CI) or aCoef (97.5% CI)	p value
Gender						
Male	197 (47.0)	222 (53.0)	0	–	0	–
Female	416 (48.5)	441 (51.5)	1.5% (− 5.1 to 8.2)	0.6086	2.0% (− 4.8 to 8.8)	0.5120
Age, years (coefficient)	–	–	0.3% (0.03–0.6)	0.0143	0.7% (0.2–1.2)	0.0017
Marital status						
Not married^1^	275 (47.0)	310 (53.0)	0	–	0	–
Married	338 (48.9)	353 (51.1)	1.9% (− 4.2 to 8.2)	0.4971	− 2.3% (− 10.2 to 5.7)	0.5243
Educational level						
None or primary or secondary education	260 (47.3)	290 (52.7)	0	–	0	–
Tertiary education	353 (48.6)	373 (51.4)	1.3% (− 5.0 to 7.7)	0.6327	0.9% (− 7.2 to 9.0)	0.8050
Work category						
Clinical staff	523 (47.1)	587 (52.9)	0	–	0	–
Non-clinical staff	90 (54.2)	76 (45.8)	7.1% (− 2.2 to 16.4)	0.0870	5.2% (− 5.0 to 15.4)	0.2506
Working experience, years (coefficient)	–	–	0.04% (− 0.3 to 0.4)	0.8182	− 0.7% (− 1.3 to (− 0.1))	0.0127
Primary place of work						
Private health facility^2^	299 (45.9)	353 (54.1)	0	–	0	–
Public health facility^3^	314 (50.3)	310 (49.7)	4.5% (− 1.8 to 10.7)	0.1106	11.6% (3.2–19.9)	0.0019
Level of primary place of work						
Tertiary health facility^4^	115 (40.4)	170 (59.6)	0	–	0	–
Primary health facility^5^ or secondary health facility^6^	498 (50.3)	493 (49.7)	9.9% (2.5–17.3)	0.0028	19.2% (10.2–28.2)	< 0.0001

Most trusted source of information about COVID-19						
	Interpersonal sources n (%)	Not Interpersonal sources^^^ n (%)	cPD (97.5% CI) or cCoef (97.5% CI)	p value	aPD (97.5% CI) or aCoef (97.5% CI)	p value
Gender						
Male	216 (51.6)	203 (48.4)	0	–	0	–
Female	420 (49.0)	437 (51.0)	− 2.5% (− 9.2 to 4.1)	0.3935	0.6% (− 6.1 to 7.6)	0.8048
Age, years (coefficient)	–	–	0.5% (0.3–0.8)	< 0.0001	0.7% (0.2–1.2)	0.0010
Marital status						
Not married^1^	266 (45.5)	319 (54.5)	0	–	0	–
Married	370 (53.6)	321 (46.4)	8.1% (1.8–14.4)	0.0039	0.3% (− 7.7 to 8.2)	0.9428
Educational level						
None or primary or secondary education	252 (45.8)	298 (54.2)	0	–	0	–
Tertiary education	384 (52.9)	342 (47.1)	7.1% (0.8–13.4)	0.0121	0.9% (− 7.2 to 8.9)	0.8121
Work category						
Clinical staff	536 (48.3)	574 (51.7)	0	–	0	–
Non-clinical staff	100 (60.2)	66 (39.8)	11.9% (2.8–21.1)	0.0034	6.2% (− 4.1 to 16.6)	0.1762
Working experience, years (coefficient)	–	–	0.3% (− 0.1 to 0.7)	0.0938	− 0.5% (− 1.1 to 0.06)	0.0449
Primary place of work						
Private health facility^2^	286 (43.9)	366 (56.1)	0	–	0	–
Public health facility^3^	350 (56.1)	274 (43.9)	12.2% (6.0–18.5)	< 0.0001	10.0% (1.6–18.3)	0.0077
Level of primary place of work						
Tertiary health facility^4^	159 (55.8)	126 (44.2)	0	–	0	–
Primary health facility^5^ or secondary health facility^6^	477 (48.1)	514 (51.9)	− 7.7% (− 15.2 to (− 0.2))	0.0220	1.5% (− 7.6 to 10.6)	0.7094

Traditional media versus non-traditional media^^^^ sources of information						
			Crude results		Adjusted results*	
Main source of information about COVID-19						
	Traditional media n (%)	Not traditional media^^^^ n (%)	cPD (97.5% CI) or cCoef (97.5% CI)	p value	aPD (97.5% CI) or aCoef (97.5% CI)	p value
Gender						
Female	326 (38.0)	531 (62.0)	0	–	0	–
Male	176 (42.0)	243 (58.0)	4.0% (− 2.6 to 10.5)	0.1756	4.3% (− 2.5 to 11.1)	0.1536
Age, years (coefficient)	–	–	0.08% (− 0.2 to 0.4)	0.5212	− 0.5% (− 0.9 to 0.03)	0.0363
Marital status						
Not married^1^	215 (36.8)	370 (63.2)	0	–	0	–
Married	287 (41.5)	404 (58.5)	4.8% (− 1.4 to 10.9)	0.0807	7.4% (− 0.5 to 15.4)	0.0367
Educational level						
Tertiary	274 (37.3)	455 (62.7)	0	–	0	–
None, primary, or secondary	231 (42.0)	316 (58.0)	4.7% (− 1.5 to 10.9)	0.0913	5.4% (− 2.5 to 13.3)	0.1274
Work category						
Clinical staff	435 (39.2)	675 (60.8)	0	–	0	–
Non-clinical staff	67 (40.4)	99 (59.6)	1.2% (− 8.0 to 10.3)	0.7740	3.3% (− 7.0 to 13.5)	0.4734
Working experience, years (coefficient)	–	–	0.4% (− 0.01 to 0.7)	0.0311	0.7% (0.2–1.3)	0.0047
Primary place of work						
Private health facility^2^	274 (42.0)	378 (58.0)	5.5% (− 0.6 to 11.6)	0.0446	4.3% (− 4.0 to 12.6)	0.2439
Public health facility^3^	228 (36.5)	396 (63.5)	0	–	0	–
Level of primary place of work						
Tertiary health facility^4^	101 (35.4)	184 (64.6)	0	–	0	–
Primary health facility^5^ or secondary health facility^6^	401 (40.5)	590 (59.5)	5.0% (− 2.2 to 12.3)	0.1203	1.9% (− 6.8 to 10.7)	0.6201

Most trusted source of information about COVID-19						
	Traditional media n (%)	Not traditional media^^ n (%)	cPD (97.5% CI) or cCoef (97.5% CI)	p value	aPD (97.5% CI) or aCoef (97.5% CI)	p value
Gender						
Female	347 (40.5)	510 (59.5)	0	–	0	–
Male	171 (40.8)	248 (59.2)	0.3% (− 6.2 to 6.9)	0.9127	3.6% (− 3.1 to 10.3)	0.2284
Age, years (coefficient)	–	–	− 0.2% (− 0.5 to 0.1)	0.0952	− 0.5% (− 1.0 to (− 0.03))	0.0180
Marital status						
Not married^1^	245 (41.9)	340 (58.1)	0	–	0	–
Married	273 (39.5)	418 (60.5)	− 2.4% (− 8.6 to 3.8)	0.3903	4.3% (− 3.5 to 12.2)	0.2149
Educational level						
Tertiary	259 (35.7)	467 (64.3)	0	–	0	–
None, primary, or secondary	259 (47.1)	291 (52.9)	11.4% (5.2–17.6)	< 0.0001	7.4% (− 0.5 to 15.4)	0.0369
Work category						
Non-clinical staff	60 (36.1)	106 (63.9)	0	–	0	–
Clinical staff	458 (41.3)	652 (58.7)	5.1% (− 3.9 to 14.1)	0.2022	0.5% (− 9.7 to 10.8)	0.9047
Working experience, years (coefficient)	–	–	0.07% (− 0.3 to 0.4)	0.6952	0.6% (0.04–1.2)	0.0160
Primary place of work						
Private health facility^2^	307 (47.1)	345 (52.9)	13.3% (7.2–19.4)	< 0.0001	3.6% (− 4.8 to 11.9)	0.3369
Public health facility^3^	211 (33.8)	413 (66.2)	0	–	0	–
Level of primary place of work						
Tertiary health facility^4^	73 (25.6)	212 (74.4)	0	–	0	–
Primary health facility^5^ or secondary health facility^6^	445 (44.9)	546 (55.1)	19.3% (12.5–26.1)	< 0.0001	14.5% (6.1–22.9)	0.0001

cPD = Crude prevalence difference. aPD = Adjusted prevalence difference. cCoef = Crude coefficient. aCoef = Adjusted coefficient.

^^^Traditional media or Internet, social media, and SMS information sources.

^^^^Interpersonal or Internet, social media, and SMS information sources.

*Adjusted for gender, age, marital status, educational level, work category, years of working experience, primary place of work, level of primary place of work.

^$^p value of overall effect.

^1^Separated or Divorced or Widowed or Never married (Single).

^2^Patent medicine vendor (PMV), Private pharmacy, Private laboratory, Private hospital or clinic, Missionary hospital.

^3^Primary health care (PHC) centre, General hospital, Federal tertiary health centre, and Federal university teaching hospital.

^4^Federal tertiary health centre and Federal university teaching hospital.

^5^PMV, Private pharmacy, Private laboratory, Private hospital or clinic, and PHC centre.

^6^Missionary hospital and General hospital.

As shown by the adjusted results, the predictors of having an interpersonal source as the main source of information about COVID-19 were: being a health worker at a primary/secondary health facility (adjusted prevalence difference (aPD) 19.2%, 97.5% CI 10.2–28.2, p < 0.0001); being a health worker at a public health facility (aPD 11.6%, 3.2–19.9, p = 0.0019); age as one year increase in age increases the probability of having an interpersonal source as the main source of information about COVID-19 by 0.7% (adjusted coefficient (aCoef) 0.7%, 97.5% CI 0.2–1.2, p = 0.0017); and working experience as one year increase in working experience reduces the probability of having an interpersonal source as the main source of information about COVID-19 by 0.7% (aCoef − 0.7%, − 1.3–(− 0.1), p = 0.0127). The predictors of having an interpersonal source as the most trusted source of information about COVID-19 were: being a health worker at a public health facility (aPD 10.0%, 1.6–18.3, p = 0.0077) and age as one year increase in age increases the probability of having an interpersonal source as the most trusted source of information about COVID-19 by 0.7% (aCoef 0.7%, 0.2–1.2, p = 0.0010).

The predictors of having traditional media as the main source of information about COVID-19 was working experience as one year increase in working experience increases the probability of having traditional media as the main source of information about COVID-19 by 0.7% (aCoef 0.7%, 0.2–1.3, p = 0.0047). The predictors of having traditional media as the most trusted source of information about COVID-19 were: being a health worker at a primary/secondary health facility (aPD 14.5%, 6.1–22.9, p = 0.0001); working experience as one year increase in working experience increases the probability of having traditional media as the most trusted source of information about COVID-19 by 0.6% (aCoef 0.6%, 0.04–1.2, p = 0.0160); and age as one year increase in age reduces the probability of having traditional media as the most trusted source of information about COVID-19 by 0.5% (aCoef 0.5%, − 1.0–(− 0.03), p = 0.0180).

### Predictors of the level of control practice about COVID-19

The associations between the level of control practice about COVID-19 and sociodemographic and background factors are presented in Table 7. As shown by the adjusted results, the predictors of good control practice about COVID-19 were: good knowledge about COVID-19 (aPD 24.0%, 17.3–30.7, p < 0.0001); good attitude towards COVID-19 (vaccination) (aPD 11.9%, 4.1–19.7, p = 0.0006); strong COVID-19 experience and perception (aPD 17.1%, 10.0–24.2, p < 0.0001); being a health worker at a tertiary health facility (aPD 10.7%, 1.0–20.4, p = 0.0130); having a tertiary education (aPD 21.7%, 14.3–29.2, p < 0.0001); and working experience as one year increase in working experience reduces the probability of having good COVID-19 control practice by 1.2% (aCoef − 1.2%, − 1.7 to (− 0.7), p < 0.0001).

**Table 7:** Association between sociodemographic and background factors and the level of COVID-19 control practice among the 1276 study participants.

Level of COVID-19 control practice^^^			Crude results		Adjusted results*	
	Good n (%)	Poor n (%)	cPD (97.5% CI) or cCoef (97.5% CI)	p value	aPD (97.5% CI) or aCoef (97.5% CI)	p value
Gender						
Male	175 (41.8)	244 (58.2)	0	–	0	–
Female	374 (43.6)	483 (56.4)	1.9% (− 4.7 to 8.5)	0.5247	2.4% (− 3.8 to 8.6)	0.3899
Age, years (coefficient)	–	–	− 0.003% (− 0.3 to 0.3)	0.9840	0.04% (− 0.4 to 0.4)	0.8199
Marital status						
Not married^1^	218 (37.3)	367 (62.7)	0	–	0	–
Married	331 (47.9)	360 (52.1)	10.6% (4.5–16.8)	0.0001	6.3% (− 1.0 to 13.5)	0.0518
Educational level						
None, primary, or secondary	167 (30.4)	383 (69.6)	0	–	0	–
Tertiary	382 (52.6)	344 (47.4)	22.3% (16.2–28.3)	< 0.0001	21.7% (14.3–29.2)	< 0.0001
Work category						
Clinical staff	479 (43.2)	631 (56.8)	0	–	0	–
Non-clinical staff	70 (42.2)	96 (57.8)	− 1.0% (− 10.2 to 8.2)	0.8108	2.6% (− 7.2 to 12.3)	0.5559
Working experience, years (coefficient)	–	–	− 0.3% (− 0.7 to 0.1)	0.0720	− 1.2% (− 1.7 to (− 0.7))	< 0.0001
Primary place of work						
Private health facility^2^	228 (35.0)	424 (65.0)	0	–	0	–
Public health facility^3^	321 (51.4)	303 (48.6)	16.5% (10.3–22.6)	< 0.0001	2.2% (− 5.6 to 10.0)	0.5275
Level of primary place of work						
Tertiary health facility^4^	158 (55.4)	127 (44.6)	16.0% (8.5–23.4)	< 0.0001	10.7% (1.0–20.4)	0.0130
Primary health facility^5^ or secondary health facility^6^	391 (39.5)	600 (60.5)	0	–	0	–
Main source of information about COVID-19				0.0734^$^		0.5146^$^
Internet, social media (whatsapp, facebook), & SMS	81 (50.3)	80 (49.7)	0	–	0	–
Traditional media (television, radio, prints)	220 (43.8)	282 (56.2)	− 6.5% (− 16.6 to 3.6)	0.1515	6.0% (− 5.9 to 17.9)	0.2572
Interpersonal^7^	248 (40.5)	365 (59.5)	− 9.9% (− 19.7 to 0.03)	0.0255	3.8% (− 8.5 to 16.2)	0.4859
Most trusted source of information about COVID-19				0.0017^$^		0.4046^$^
Internet, social media (whatsapp, facebook), & SMS	71 (58.2)	51 (41.8)	0	–	0	–
Traditional media (television, radio, prints)	216 (41.7)	302 (58.3)	− 16.5% (− 27.6 to (− 5.4))	0.0009	− 7.8% (− 21.1 to 5.4)	0.1851
Interpersonal^7^	262 (41.2)	374 (58.8)	− 17.0% (− 27.9 to (− 6.1))	0.0005	− 7.3% (− 21.1 to 6.4)	0.2311
Level of knowledge about COVID-19^8^						
Poor	220 (29.9)	516 (70.1)	0	–	0	–
Good	329 (60.9)	211 (39.1)	31.0% (25.0–37.1)	< 0.0001	24.0% (17.3–30.7)	< 0.0001
Level of attitude towards COVID-19 (vaccination)^9^						
Poor	51 (21.3)	188 (78.7)	0	–	0	–
Good	498 (48.0)	539 (52.0)	26.7% (19.8–33.6)	< 0.0001	11.9% (4.1–19.7)	0.0006
Extent of COVID-19 experience and perception^D,S^						
Not strong	188 (33.3)	376 (66.7)	0	–	0	–
Strong	361 (50.7)	351 (49.3)	17.4% (11.2–23.5)	< 0.0001	17.1% (10.0–24.2)	< 0.0001

cPD = Crude prevalence difference. aPD = Adjusted prevalence difference. cCoef = Crude coefficient. aCoef = Adjusted coefficient.

^Practice score of ≥ 75% of the highest attainable score of 24 was good practice and < 75% was poor practice.

*Adjusted for level of knowledge about COVID-19; level of attitude towards COVID-19 & COVID-19 vaccination; extent of COVID-19 experience and perception; source of information about COVID-19 (main source and most trusted source of information about COVID-19); sociodemographic characteristics (gender, age, marital status, educational level); and work related attributes (work category, years of working experience, primary place of work, level of primary place of work).

^$^p value of overall effect.

^1^Separated or Divorced or Widowed or Never married (Single).

^2^Patent medicine vendor (PMV), Private pharmacy, Private laboratory, Private hospital or clinic, Missionary hospital.

^3^Primary health care (PHC) centre, General hospital, Federal tertiary health centre, and Federal university teaching hospital.

^4^Federal tertiary health centre and Federal university teaching hospital.

^5^PMV, Private pharmacy, Private laboratory, Private hospital or clinic, and PHC centre.

^6^Missionary hospital and General hospital.

^7^Relatives/friends, health workers, place of work, place of worship etc.

^8^Knowledge score of < 75% of the highest attainable score of 44 was poor knowledge and ≥ 75% was good knowledge.

^9^Attitude score of < 75% of the highest attainable score of 80 was poor attitude and ≥ 75% was good attitude.

^D^Disease risk perception.

^S^Perception score of < 50% of the highest attainable score of 32 was not strong perception and ≥ 50% was strong perception.

## Discussion

This study explored the COVID-19 information sources, knowledge, attitude, and control practices, and the predictors among health workers during the COVID-19 pandemic in Ebonyi state, Nigeria. The main study findings include: the overall commonest source of information was health workers followed by radio, television, and family members/relatives/friends; interpersonal sources like health workers was the main and most trusted source of information for majority of the participants followed by traditional media such as radio; 42.3% had good knowledge about COVID-19, 81.3% had good attitude towards COVID-19 (vaccination), and 43.0% had good control practice about COVID-19.

In this study: the predictors of having an interpersonal source as the main source of information about COVID-19 were being a health worker at a primary/secondary health facility, being a health worker at a public health facility, increase in age, and increase in years of working experience; the predictors of having an interpersonal source as the most trusted source of information about COVID-19 were being a health worker at a public health facility and increase in age; the predictors of having traditional media as the main source of information about COVID-19 was increase in years of working experience; the predictors of having traditional media as the most trusted source of information about COVID-19 were being a health worker at a primary/secondary health facility, increase in years of working experience, and decrease in age; and the predictors of good control practice about COVID-19 were good knowledge about COVID-19, good attitude towards COVID-19 (vaccination), strong COVID-19 experience and perception, being a health worker at a tertiary health facility, having a tertiary education, and decrease in years of working experience.

In our study, health workers was the commonest source of information about COVID-19 (and radio was the second commonest). In comparison, the commonest source of information about COVID-19 was ministry of health (website) in studies in Saudi^7,12^ and scientific literature in Venezuela^14^. In our study, interpersonal sources like health workers was the main and most trusted source of information for majority of the participants (and traditional media such as radio was the second main and most trusted source). In comparison, the main information source was social media in a study in Nepal^9^ and Yemen^11^; ministry of health in Saudi^10^; internet websites in Jordan^13^; the World Health Organization^15^ and radio/television^6^ in Ethiopia.

In contrast to the other previous studies, the fact that interpersonal sources like health workers was the commonest, main, and most trusted source of information for majority of the participants has some important policy implications. Unlike the other studies, our study was not only online but also offline and among the entire populations/categories of health workers in the formal and informal health sectors and in both urban/semi-urban and rural areas. As a result, our study perhaps had relatively more proportion of lay/lower cadre health workers and/or health workers in rural/semi-urban who had no access or poor access to the internet and social media. This made interpersonal sources of information to be relatively very popular and this very much reflects the context of the study setting. This logic is supported by our study finding that being a health worker at a primary/secondary health facility (mostly found in remote rural areas) was a predictor of interpersonal source as the main source of information about COVID-19 compared to being a health worker at a tertiary health facility (found in the state capital). Our finding also indicate that health workers are not only health opinion leaders and important source of health information for the general public but also for other health workers especially in poor resource settings. In the study in Venezuela, colleagues/other health workers was the second commonest source of information after scientific literature even though the study was only online^14^. It is good to note that many of the previous studies did not measure/report on the use of health workers or interpersonal sources of information. Subsequent studies among health workers in poor resource settings should include the use of interpersonal sources of information (health workers etc.) in the evaluation of (health emergency) information sources.

The aforementioned explanation/reason can also be used to explain our findings that radio was the second commonest, main, and most trusted source of information for majority of the participants. Compared to the other modern/mass media, radio is more accessible and affordable with more geographical coverage including the remote rural areas. As a result, the fact that radio was the second most popular source of information was a reflection of the prevailing context of the study setting. Radio is a major channel that is always used by Ebonyi state government for information dissemination and it is very popular across the entire state. Perhaps the information from radio is more or less mainly from the state government. Thus, the fact that radio was the second commonest, main, and most trusted source of information about COVID-19 in our study, coupled with the fact that government sources (ministry of health) was the commonest and main source of information in the studies in Saudi^7,10,12^, emphasises the usefulness of government information channels during health emergencies and calls for government to take-charge of heath information disseminating during health emergencies especially when there is a lot of misinformation/disinformation and conspiracy theories as observed during the COVID-19 pandemic. Subsequent qualitative studies on the factors that influence health workers’ confidence in government as a source of health information during health emergencies are recommended.

In our study, 42.3% had good knowledge about COVID-19, 81.3% had good attitude towards COVID-19 (vaccination), and 43.0% had good control practice about COVID-19. In comparison, higher prevalence of: 83.7% had good knowledge and 77.6% had good preventive practice (although similar proportion of 78.9% had positive attitude) about COVID-19^4^ and 88.6% had good knowledge and 81.4% had good preventive practice about COVID-19^8^ in studies in Nigeria. However, another study in Nigeria reported lower prevalence of good attitude of 58.3% and good practice of 38.6% and higher prevalence of good knowledge of 59.1%^5^. Similarly, higher and lower prevalence were reported by foreign studies: higher prevalence of good knowledge of 48.2% and lower prevalence of good attitude of 33.8% in a study in Saudi^7^; higher prevalence of adequate knowledge of 76.0% and appropriate preventive practice of 78.6% and lower prevalence of positive attitude of 54.7% in Nepal^9^; higher prevalence of adequate knowledge of 67.8% and appropriate practice of 80.2% and lower prevalence of positive attitude of 72.2% in Saudi^10^; higher prevalence of adequate knowledge of 69.8% and good practice of 87.7% and slightly higher prevalence of positive attitude of 85.1% in Yemen^11^; and higher prevalence of good knowledge of 81.4%, good attitude of 87.1% and satisfactory practice of 77.9% in Jordan^13^. Higher prevalence (especially of good knowledge and good practice) and lower prevalence (especially of good/positive attitude) were also reported by other studies in Venezuela^14^, Ethiopia^6,15^, Sierra Leone^16^, and Pakistan^17^.

The comparatively higher/lower values in the other previous studies could perhaps be explained by contextual and timing differences between those studies and our study. Previous evidence has shown time and context-specific trends in COVID-19 risk perception^20^ and COVID-19 knowledge, attitude, and preventive practice^21,22^. Unlike our study: most of the previous studies were only online among privileged heath workers who largely had good internet access; involved limited categories of health workers; were implemented during the initial waves of the pandemic when uncertainties, confusion, fear, and anxiety were more prevalent; and some of those studies were conducted in settings with higher burden of the pandemic. These contexts means the health workers in those studies were of different sociodemographic background and relatively had higher/more privileged professional attributes and socioeconomic/educational status which could have accounted for the higher prevalence of good knowledge and good practice in those studies. Higher socioeconomic and educational status have been associated with higher COVID-19 knowledge and preventive practice^22^.

The largely lower prevalence of good/positive attitude towards COVID-19 in those studies could also be explained by the more widespread negative perceptions during the initial waves of the pandemic when there were much uncertainties, confusion, fear, and anxiety amidst the unprecedented misinformation, disinformation, and conspiracy theories. In addition, there were fewer number of COVID-19 cases/related-deaths and limited or no actual COVID-19 vaccines/vaccination during the initial waves of the pandemic when most of the previous studies were conducted. As a result, perhaps fewer participants in those studies had direct/indirect experience of COVID-19 and more of the participants had limited or no real experiences of the importance, safety/side-effects, and effectiveness of COVID-19 vaccination/vaccines and were perhaps more prone to the adverse influence of the misinformation/disinformation which could have more adversely affected their perceptions regarding COVID-19 and COVID-19 vaccines/vaccination. Subsequent studies, especially qualitative studies, are needed to provide more insights on how interactions between misinformation/disinformation/conspiracy theories and real direct/indirect experiences influence perceptions and behaviours/practices regarding COVID-19/health emergencies.

We did not identify any relevant and comparable studies to appropriately compare our findings regarding the predictors of having an interpersonal source as the main/most trusted source of information about COVID-19 and of having traditional media as the main/most trusted source of information about COVID-19. However, our findings provide the evidence that professional/work-related attributes and sociodemographic characteristics were important determinants of the health workers’ use of and confidence in COVID-19 information sources. Considering the fact that interpersonal source (such as health workers) was the commonest, main, and most trusted source of information about COVID-19 (followed by traditional media (such as radio)) in our study, the identified predictors indicate professional and sociodemographic factors to be considered in designing and implementing subsequent COVID-19/health emergencies’ health information tailored to health workers in Ebonyi state/Nigeria and other similar settings. Subsequent studies on COVID-19/health emergencies should not only aim to describe the use of information sources but should extensively assess health workers’ use of and confidence in information sources and the predictors.

In our study, the predictors of good control practice about COVID-19 were good knowledge about COVID-19, good attitude towards COVID-19 (vaccination), strong COVID-19 experience and perception, being a health worker at a tertiary health facility, having a tertiary education, and decrease in years of working experience. It is worth noting that the main and most trusted source of information about COVID-19 were not predictors. Similarly, the predictor of COVID-19 preventive practice in other studies were: good knowledge of COVID-19 (two studies in Nigeria)^4,5^, higher educational level (Nepal, Saudi)^9,10^, male gender (Saudi)^10^ and 5 or more years of working experience in Ethiopia^6^. Also, COVID-19 information sources were not predictors in a study in Nepal^9^. However, COVID-19 information source was a predictor in a study in Ethiopia^6^. Also, working in a primary health facility was a predictor in one of the studies in Nigeria^5^ in contrast to being a health worker at a tertiary health facility in our study. In addition, good/positive attitude towards COVID-19 were not predictors in the studies in Nigeria^4,5^ and adequate knowledge was not a predictor in Ethiopia^6^.

The contrasting findings regarding predictors could be due to the differences in timing of the studies and contextual factors and differences in the definitions, measurements, and categorization of predictor and outcome variables. Perhaps the higher confidence level of 97.5% that we used to correct for multiple comparisons reduced the number of predictors identified in our study. However, these predictors should be prioritised in the development of context-specific and tailored policy interventions to increase subsequent control practice about COVID-19 and similar health emergencies.

Reporting bias was a potential limitation in this study which was questionnaire-based study that relied on participants expressing their perceptions and control practices regarding COVID-19. The sensitive nature of the COVID-19 pandemic and the national/global response to control the pandemic perhaps increased the tendency for some respondents to exaggerate desirable perceptions and practices and underestimate undesirable perceptions and practices. To minimise this bias, the anonymous and confidential nature of the questionnaire survey were properly explained and emphasized to the participants. This study was also prone to selection bias because of the convenience and snowballing sampling techniques employed for the selection of study participants.

This study had many strengths. It was both online and offline and involved the entire populations and categories of health workers (in the formal and informal health sectors) in both rural and urban/semi-urban areas in Ebonyi state. Thus, the findings of this study are more generalisable to the general population of health workers in the state and other parts of Nigeria, including other less privileged settings with limited internet access. In addition, the outcome measures and the potential covariates were pre-specified in the study protocol which was prospectively registered and prospectively submitted to a peer-review journal before the implementation of this study started.

## Conclusions

The commonest source of information about COVID-19 and the main and most trusted source of information for majority of the health workers during the COVID-19 pandemic in Ebonyi state, Nigeria was interpersonal sources such as health workers followed by traditional media such as radio. There was low knowledge as only less than half had good knowledge about COVID-19, satisfactory attitude as four-fifth had good attitude towards COVID-19 (vaccination), and poor practice as only less than half had good control practice about COVID-19. The most important predictors of the main and most trusted sources of information about COVID were primary place of work (public/private facility), level of primary place of work (primary/secondary versus tertiary facility), age, and years of working experience. Good knowledge about COVID-19, good attitude towards COVID-19 (vaccination), strong COVID-19 experience and perception, being a health worker at a tertiary health facility, having a tertiary education, and decrease in years of working experience were strong predictors of good control practice about COVID-19.

Subsequent policy actions regarding COVID-19 and similar health emergencies in Ebonyi state and Nigeria, and other similar settings, should prioritise the identified commonest, main, and most trusted information sources, their predictors, and the predictors of good control practice about COVID-19 in the strategies to optimise emergency health information dissemination and use of control measures by health workers respectively. Later studies should explore the extent of two-way communications, and the determinants, in the health emergency information dissemination process and the effects on the use of control measures by health workers.

## Data Availability

The datasets used and/or analysed during the current study are available from the corresponding author on reasonable request.
